# Assisted Dying Makes Us Think Differently About Care

**DOI:** 10.1111/hex.70723

**Published:** 2026-07-16

**Authors:** Patrick Bolton, Ben P. White, Julian Huppert, Ann Dadich

**Affiliations:** ^1^ Prince of Wales Hospital South Eastern Sydney Local Health District Randwick Australia; ^2^ Australian Centre for Health Law Research Queensland University of Technology George Street Brisbane Australia; ^3^ Jesus College University of Cambridge Cambridge United Kingdom of Great Britain and Northern Ireland; ^4^ School of Business Western Sydney University Parramatta NSW Australia

**Keywords:** assisted suicide, decision‐making, euthanasia, family‐centred care, health personnel attitudes, palliative care, patient autonomy, patient‐centred care, professional–patient relations, terminal care

## Abstract

Assisted dying (AD) is increasingly available as a legal end‐of‐life option in wealthy nations. Literature on AD considers its operation and the experience of clinicians, people receiving AD, and their loved ones. It is inward‐looking. The nature of AD suggests it focuses less on the technical aspects of care and more on its humanity. Accordingly, AD offers lessons for healthcare more broadly—specifically, the importance of patient‐ and family‐centred care, respect for patient wishes, and relational care.

**Lived Experience:**

JH is a family member of a person who received assisted dying. The vignettes are AD's lived experience.

## 1 Introduction

Assisted dying (AD) is increasingly available as a legal end‐of‐life option in wealthy nations. Literature on AD considers its operation and the experience of clinicians, people receiving AD, and their loved ones. It is inward‐looking. The nature of AD suggests it focuses less on the technical aspects of care and more on its humanity. Accordingly, AD provides lessons for the healthcare system because it is a technically simple process with a complex emotional, social, and legal penumbra. Complexity arises because of perceived tensions between AD and the foundational percept to ‘first do no harm’. It thereby facilitates consideration of medicine beyond the technical.

While the opportunity for AD to inform the practice of medicine has been identified, this opportunity has not been pursued. AD providers have described it as ‘special’ [1], enabling ‘a beautiful death’ [2], and an ‘honour to provide’ [3], simultaneously noting it can create tensions with other clinicians. AD emphasises relationships, particularly with recipients and their families but also other clinicians.

This article explores what AD practice reveals about the deeper workings of medicine. Three key interconnected characteristics, arising across clinical experiences and jurisdictions, are considered to establish how AD challenges assumptions about care, responsibility, and professional identity. Together, these key characteristics illuminate opportunities for medicine to reflect on and perhaps reshape its broader practices. The article concludes by considering how lessons from AD might strengthen healthcare more widely.

## 2 Key Characteristics

### 2.1 Person‐Centred Care

AD highlights the importance of patient‐ and family‐centred care, where dignity, values, and personal meaning take precedence. Medicine and society's pursuit of long life can overshadow the equally vital need for a person's final moments to be marked by dignity, comfort, and meaning. When medical control is privileged over personal meaning, the essence of what it means to care can be missed. It might mean holding a hand, facilitating reconciliation, or simply being there. To truly care is to listen without rushing, to honour the person's values and wishes, as well as those of their grieving family members, and to create space for meaningful connection, even in silence. While technical expertise is needed to guide treatment, more is needed—namely, empathy.

A good death is not defined solely by the absence of pain or the presence of medical intervention—it is shaped by the opportunity to say goodbye, to reconcile, to be heard, and to be held [4]. When life extension is prioritised without equal attention to these human elements, individuals can be reduced to clinical cases, rather than recognising them as whole people nearing the end of their journey.

Actively ending a person's life through the AD process is one of the most intimate and socially meaningful things one human can do with another. It requires clinicians to be not only technically skilled but also emotionally attuned—to balance science with soul. Only then can care be offered that is holistic, honouring the body and the spirit as life draws to a close. Supporting a good death demands presence, courage, and a willingness to honour the person's voice.

### 2.2 Respect for Patient Wishes

AD encourages open conversations about death, reframing it not as a failure but as a natural and potentially peaceful conclusion. This shift fosters trust between patients and clinicians, and promotes a culture where emotional, spiritual, and existential needs are addressed alongside physical ones. This is often therapeutic.

End‐of‐life care often fails to align with the values and wishes of people who are dying. Patients are often more open to dying than their doctors. Physicians would not choose for themselves some of the treatments they offer patients [5]. It is hard, perhaps impossible, to think about what it is like to suffer when one is not suffering and to suffer without a clear end—this might skew clinical assessment.

These reflections were informed by the death of one author's father. He hated the hospital and wanted to be home. Despite the best efforts of his family to enable a home death, the doctor delayed his hospital discharge, saying he was likely to die while travelling home. For the family, this was not the point—the point was to provide the man with what he wanted. This was not to be. While the doctor was not necessarily focused on prolonging life, he was focused on clinical risk, on avoiding the possibility of death in transit. But for the author's father, the risk was worth it. He wished to die in the comfort of his own home, surrounded by familiar sounds, smells, and people. The doctor's decision overlooked the emotional and existential significance of place, autonomy, and dignity.

In most medical contexts, the person giving consent is passive: ‘I'll go and consent that patient’—something that is done *to* the patient. The importance of the AD decision and its social and legal contexts demand consent as an active act by the person. The legal structure around AD demands that a person's choice is robustly tested and scrutinised through the application process and external review. To be sure of this decision, clinicians must take a different approach to informed consent. Time needs to be taken to explore what is important to the person and support the person to make a choice that aligns with their identified values.

### 2.3 Relational Care Versus Transactional Care

What makes for a good death is not clinical. It is personal. It is relational. Palliative care as a discipline has known this for a long time. This has also become clear to the people involved in AD. This is particularly because the technical skills required to provide AD are not especially challenging. Instead, providing safe, high‐quality AD engages these ‘other’ domains. A challenge for medicine is that sometimes these other skills and considerations are not seen as important or valuable. Caring for someone at the end of life transcends clinical precision; it is a deeply human act that calls for compassion, presence and respect.

Supporting a good death requires a shift in mindset—from doing *to* the patient, to being *with* the patient. It calls for humility, empathy, and the courage to acknowledge that death is not a failure but a natural part of life. Reclaiming the art of dying well is not about abandoning science but about integrating it with compassion and presence.

## 3 Implications for Medicine

When my mother was dying, the ‘‐ologists’ brought their expertise, their precision, and their protocols. This served my family—we were desperate for glimpses of hope. However, expertise, precision and protocols were unlikely to benefit my mother. What she probably needed in those final days was not another test or treatment plan. She needed someone to sit beside her, to speak gently, and to acknowledge her courage. It was a palliative care doctor whose words offered comfort—who helped my family members recognise the need to let go. And it was the graveyard‐shift nurse who gently extended time and compassion, as we watched my mother breathe. These moments of care were not technical; they were tender. Her death taught me that, while clinical precision can sustain life, it is compassion that sustains humanity.

End‐of‐life care must be more than a series of interventions; it must be a practice of presence. When we care with both skill and soul, the person and their family members are honoured, not just the patient, and death is allowed to be, not only inevitable, but meaningful:

My father's final hours were spent in a hospital he disliked, not because it was necessary but because the system defaulted to safety over soul.

A good death is not defined by clinical outcomes but by the alignment of care with the person's values. AD compels clinicians to think differently about care.

## 4 Conclusion

All areas of medicine are complex. Outcomes are uncertain—a treatment can only be said to be probable, not certain. Decisions based on populations (which are impersonal) are applied to individuals. AD is simpler in that the desired outcome from a simplistic perspective is clear—the patient ceases to live. It is not technically challenging to provide.

AD's simplicity creates more space for the non‐clinical. Furthermore, AD forces the non‐clinical to the fore in a way that might be obscured if it had to compete for attention with complex medical decisions. AD invites a broader, more courageous conversation about what it means to die well. It challenges clinicians to move beyond a one‐size‐fits‐all approach to end‐of‐life care and instead embrace nuance, choice and the diversity of human experience.

AD teaches us that care must be as individual as the lives we lead. It calls for humility, dialogue, and a willingness to see illness and death not only as a clinical event but also as a profoundly human one. In doing so, it can help reshape medicine into a practice that honours patient experience with grace.

AD presents an opportunity for medicine to pause and refocus on the person at the centre of care. It challenges clinicians to engage with autonomy, compassion, and the ethics of care in profound ways. It teaches that medicine is not only about intervention but also about listening, respecting choice, and recognising when the most humane act may be to let go. AD invites the medical system to reflect on its core purpose—not just to preserve life but to honour its quality and its end.

## Author Contributions

**Patrick Bolton:** conceptualisation, writing – original draft, writing – review and editing, methodology, project administration. **Ben P. White:** conceptualisation, methodology, writing – original draft, writing – review and editing. **Julian Huppert:** conceptualisation, methodology, writing – original draft, writing – review and editing. **Ann Dadich:** conceptualisation, methodology, writing – original draft, writing – review and editing.

## Funding

The authors have nothing to report.

## Ethics Statement

The authors have nothing to report.

## Consent

The authors have nothing to report.

## Conflicts of Interest

Conjoint Associate Professor Patrick Bolton: Conjoint Associate Professor Bolton is a publicly employed AD practitioner and medical lead for the South Eastern Sydney Local Health District Voluntary Assisted Dying Liaison Service. He has received funding from The Wicking Trust to conduct research pertaining to assisted dying.

Distinguished Professor Ben P. White: Distinguished Professor received funding from the Australian Research Council, the National Health and Medical Research Council, Commonwealth and state governments, and philanthropic organisations for research and training about the law, policy and practice relating to end‐of‐life care. In relation to voluntary assisted dying, he (with colleagues) has been engaged by the Victorian, Western Australian and Queensland governments to design and provide the legislatively mandated training for health practitioners involved in voluntary assisted dying in those states. He was appointed as an Expert Legal Advisor to the Legal and Constitutional Affairs Committee of the Legislative Assembly of the Northern Territory for its report on voluntary assisted dying and (with colleagues) developed the accompanying drafting instructions. He was also engaged (with colleagues) to provide a research report to support the Western Australian review of the voluntary assisted dying laws. He is a member of the Tasmanian Panel for the review of the End‐of‐Life Choices (Voluntary Assisted Dying) Act 2021. He (with Lindy Willmott) developed a model bill for voluntary assisted dying for parliaments to consider. Ben is a recipient of an Australian Research Council Future Fellowship (project number FT190100410: Enhancing End‐of‐Life Decision‐Making: Optimal Regulation of Voluntary Assisted Dying) funded by the Australian Government. He is also a Chief Investigator on a current Australian Research Council Linkage Project on voluntary assisted dying (partnering with Voluntary Assisted Dying [Review] Boards and/or Departments of Health in five Australian States).

Dr Julian Huppert: Dr Huppert is the Chair of two National Health Service Ethics Committees and a Member of the Nuffield Council on Bioethics. He is also a Patron of Humanists UK and has spoken in the Houses of Commons and Lords about Assisted Dying. His mother died in New South Wales on August 6, 2024, benefiting from the VAD laws in NSW.

Professor Ann Dadich: Ann Dadich is the Deputy Director of the SPHERE Knowledge Translation Platform and has received funding from The Wicking Trust to conduct research pertaining to assisted dying.

## Permission to Reproduce Material From Other Sources

Not applicable.

## Data Availability

Data sharing is not applicable to this article as no datasets were generated or analysed during the current study. No data were presented in this article.
